# MEDICAL COMPLICATIONS IN CHILDREN WITH CENTRAL NERVOUS SYSTEM INJURIES DURING INPATIENT REHABILITATION

**DOI:** 10.2340/jrm.v58.44907

**Published:** 2026-06-15

**Authors:** Kristine Marie M. VEGE, Grethe MÅNUM, Katharina S. SUNNERHAGEN, Frank BECKER

**Affiliations:** 1 Department of Research, Sunnaas Rehabilitation Hospital, Nesoddtangen; 2 Institute of Clinical Medicine, Faculty of Medicine, University of Oslo, Oslo; 3 Beitostølen Healthsports Center, Beitostølen; 4 Institute of Health and Society, Faculty of Medicine, University of Oslo, Oslo, Norway; 5 Institute of Neuroscience and Physiology, University of Gothenburg, Gothenburg, Sweden

**Keywords:** children, youth, spinal cord injury, acquired brain injury, medical complications, rehabilitation

## Abstract

**Objective:**

To investigate the occurrence and consequences of medical complications during inpatient rehabilitation of children with central nervous system (CNS) injury.

**Design:**

A prospective observational study conducted at a tertiary rehabilitation centre.

**Subjects:**

Participants aged 2 to 17 years with acquired CNS injury requiring multidisciplinary inpatient rehabilitation.

**Methods:**

Data were collected from medical charts and questionnaires on admission and discharge, assessing predefined complications and associated impacts on the rehabilitation process.

**Results:**

Of the 64 participants, 59 experienced a total of 183 instances of medical complications, comprising 19 distinct complication types. Most common were sleep disturbances, pain, constipation, anaemia, infections, spasticity, and malnutrition. No significant correlation was observed between the occurrence of complications and age, gender, or diagnosis group. In total, 74% of the complications were found to have no impact on the rehabilitation process; however, malnutrition, infections, and spasticity could notably lead to interrupted or prolonged stays, altered rehabilitation goals, and worsened functional prognoses.

**Conclusion:**

Medical complications frequently occur during inpatient rehabilitation. While many have no significant consequences, malnutrition, infections, and spasticity in particular warrant further attention clinically and in future research.

## LAY ABSTRACT

We studied medical complications in children admitted to specialized rehabilitation after acquired injury to the central nervous system; 64 patients aged 2–17 years participated. Medical complications that were ongoing or occurring during the stay were registered. Together with the responsible physicians, consequences of the complications were also assessed. In total, 59 of the 64 patients experienced medical complications. The most common were sleep disturbances, pain, constipation, anaemia, infections, spasticity, and malnutrition. A total of 183 complications were registered. Of these, 75% were assessed to not have any consequences for the rehabilitation process. Malnutrition, infections, and spasticity, however, stood out and would often lead to interrupted or prolonged stays, altered rehabilitation goals, and worsened functional prognoses.

Injuries to the central nervous system (CNS) can have serious consequences, as the CNS regulates essential functions (1). In addition to direct consequences of the injury itself – whether brain injury or spinal cord injury (SCI) – additional complications may arise, impacting patient rehabilitation in both the acute and subacute phases (2). Consequences occurring long after the initial injury have been reported in previous work by the authors (3) and others (4).

Certain areas of paediatric rehabilitation remain under-researched compared with adult populations. There exist several studies on CNS injuries of varying aetiologies in adults, comprehensively evaluating the rehabilitation process and potential complications. Such studies highlight the frequent occurrence of medical complications and emphasize the importance of early detection in prevention and management, reducing the risk of adverse outcomes such as functional deterioration as a direct result of the complication or due to interruptions to the rehabilitation process (5–10). However, there is a paucity of broad-scope studies focusing exclusively on complications following CNS injury in children. Several studies examine a wide range of medical complications separately, often regarding 1 specific CNS-related aetiology at a time (2).

A medical complication can be defined as a secondary disease/condition that develops during the course of a primary condition (either as a result of it or from independent causes) rendering the existing illness more difficult to treat (11). Complications can range from general phenomena such as pain, sleep disturbances, nausea, and weight changes (12–14), to more specific conditions such as endocrine disorders, heterotopic ossification, and pressure ulcers (15–17). Thus, the spectrum of possible complications is wide, and their capabilities for adverse effects underscore the importance of knowledge among rehabilitation specialists.

The primary objectives of critical care are to restore health and prevent complications (18). These goals remain central throughout the inpatient rehabilitation process, which is characterized by systematic efforts to enhance functional capacity, promote independence, and support the re-establishment of a meaningful life following disability (19, 20). Ideally, rehabilitation is an individualized and goal-oriented process delivered by a multidisciplinary team (MDT) providing targeted interventions and comprehensive therapeutic support (21). However, medical complications may disrupt this process, potentially leading to a range of adverse clinical and functional outcomes.

Complications comprise several aspects. Pain, constipation, and poor sleep may exacerbate physical discomfort in the patient. Members of the rehabilitation team may be affected where complications negatively impact therapy sessions and patient availability for therapy. Hospitals may also be economically impacted in cases where complications necessitate longer stays or increased resource allocation; one such example of this is postponed active rehabilitation due to infection. As many complications are preventable (22), increasing knowledge and awareness is key.

Despite the clinical relevance, there remains a significant knowledge gap on the occurrence and impact of medical complications during inpatient rehabilitation of children with CNS injuries. The aim of this prospective study was therefore to investigate the frequency of such complications and their impact on the rehabilitation process. Through identifying and characterizing these complications, this study seeks to contribute to improved clinical awareness and ultimately reduce adverse outcomes. Based on the findings of previous studies (2) and clinical experience, a high occurrence of complications during inpatient rehabilitation of this patient population was hypothesized.

## MATERIALS AND METHODS

### Study design, context, and ethics

This was a prospective observational study conducted at the Sunnaas Rehabilitation Hospital (SRH), a tertiary rehabilitation centre in the south-east of Norway. The study inclusion criteria were acquired CNS injury; an age at injury of between 2 and 17 years; and consequences of the injury necessitating multidisciplinary inpatient rehabilitation.

Approximately 3.1 million people reside in south-eastern Norway, 620,000 of whom are children. All individuals aged 0–18 years with acquired CNS injury in this region are offered specialized rehabilitation regardless of insurance status and socioeconomic classification. The Regional Health Trust established a patient care protocol according to which such patients are transferred to the SRH as soon as they are considered medically stable (i.e., independent respiratory function and infections under control). All hospitals with acute care facilities are aware of this protocol and refer such patients to the SRH. SRH offers specialized patient-centred rehabilitation programmes. Each patient is assigned an MDT consisting of a nurse, physician, physical therapist, occupational therapist, psychologist, social worker, speech therapist/special needs teacher, dietitian, and team coordinator. The rehabilitation process is individualized, goal-driven, and includes meetings with the MDT. Patient caregivers are present during the hospital stay. The duration of the stay is individualized according to each patient’s rehabilitation needs.

This project was approved by the Norwegian Regional Committee for Medical and Health Research Ethics (approval number 483399). Informed consent was required by the ethical committee from both parents. Consent was obtained from both parents, except in cases of single parents. Patients from the age of 16 years provided consent themselves.

A user panel was consulted in the development of this project, consisting of 4 individuals. Two of these were former patients (< 18 years); 2 were the next of kin (1 father and 1 sister) of paediatric patients formerly admitted to the SRH.

### Participants

Patients were recruited prospectively by a project member (a rehabilitation physician) from December 2022 to December 2024. Information concerning the study was provided to patient caregivers shortly after admission.

The CNS injuries of the patients included in this study varied in localization and severity; however, all were at a level necessitating inpatient multidisciplinary rehabilitation according to established regional clinical protocols. Patients were divided into 6 subgroups regarding diagnoses. The acquired brain injuries were categorized according to aetiology as traumatic brain injury (TBI) with severity grades based on the Glasgow Coma Scale (GCS) (23), stroke (haemorrhagic and ischaemic), cerebral infection or inflammation (meningitis, encephalitis, and demyelination), cerebral neoplasms, and anoxic brain injury. Due to the low prevalence of SCIs, they were treated as 1 group regardless of aetiology (traumatic, neoplastic, vascular). SCI patients were classified using the American Spinal Injury Association (ASIA) Impairment Scale (24).

### Data

**Fig. 1:**
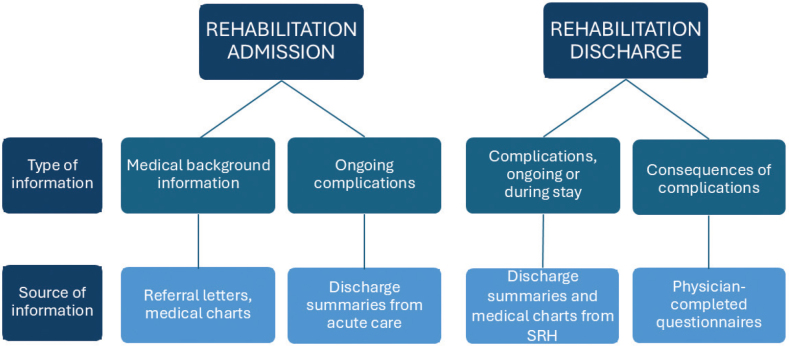
Data collection: overview of which types of data were collected at which data points and from which sources. SRH: Sunnaas Rehabilitation Hospital.

After inclusion, a study associate recorded patient background information on injury, complications ongoing upon admission for inpatient rehabilitation at SRH (using a questionnaire), and whether the complication required examination and/or treatment before admission to inpatient rehabilitation. This information was retrieved from patient medical records, including discharge summaries from acute care, referral letters, and other relevant documents. The data registration process is illustrated in Fig. 1.

Complications were categorized according to a predefined list of 32 complications, based on relevant literature (5, 9, 10, 25) (Table I). This list was also discussed and validated with the user panel. Anaemia, plus endocrine and electrolyte disturbances were recorded when blood test results deviated from the normal range. Epilepsy was registered when the patient had experienced 1 or more epileptic seizures and was receiving antiepileptic treatment on admission. All grades of pressure ulcers were classified as complications, as they require medical attention regardless of severity. Sleep disturbances were recorded when the patient received drug treatment for this problem. Spasticity was registered when clinical examination demonstrated a velocity-dependent resistance that was considered troublesome for the patient.

**Table I:** Complete list of complications

List of complications
Epilepsy, pain, infections, cardiac rhythm disorders, high blood pressure (autonomic dysregulation), low blood pressure (orthostatic hypotension), blood clots in the lungs or legs, excessive mucus in the lungs, anaemia, fractures, osteoporosis, scoliosis, heterotopic ossifications, hip dislocation, endocrine disruptions, hydrocephalus, spasticity, temperature dysregulation, stroke (brain), syringomyelia, constipation, diarrhoea, nausea/vomiting, electrolyte disturbances, dehydration, malnutrition, overweight, urinary stone formation, urinary tract problems, bone flap problems, pressure ulcers, sleep disorders, others

Similar details were recorded following discharge from inpatient rehabilitation (see Fig. 1). These included any complications present during inpatient rehabilitation, when these complications occurred/resolved, and any required examinations and/or treatments. The questionnaire used was similar to that utilized at the admission checkpoint. The study associate screened the patient medical charts prospectively for the complications that occurred during hospital stay and the medications administered.

The consequences of each complication were assessed with the patient’s assigned rehabilitation physician according to a predefined list (Table II) and registered on a separate form, retrospectively. During this session, the physicians were also asked to assess to what degree each complication impacted the overall rehabilitation process, characterized as “not at all”, “little”, “to some degree”, and “largely”. In this context, the overall rehabilitation process is intended to encompass the patient’s entire inpatient stay, including progress, achievement of rehabilitation goals, and any disturbing or impeding factors. The physicians were either specialists in physical medicine and rehabilitation, training to become such, or other medical specialists with years of rehabilitation experience. The physicians were informed of the study aims and that similar data were recorded on admission. The same study associate performed both data registrations: on admission, and, alongside the physicians, after discharge. The registration forms used on admission and discharge can be found in Appendix S1.

**Table II:** Consequences of medical complications assessed after discharge

Possible consequences
Cancelled therapy sessions Shorter therapy sessions Fewer planned sessions Extra observational measures Increased physician workload Increased workload on other employees Prolonged or interrupted stay Delayed rehabilitation process Altered process/rehabilitation goals Less improvement than expected

Additional data were obtained from the SRH electronic medical records. These included age, gender, primary diagnosis, other diagnoses, date of injury, length of stay (LOS), days of leave, curtailed stay, and discharge destination. The date of injury was defined as the exact date of traumatic injuries or the onset of illness (e.g., stroke). Where the exact onset of the illness was unclear, the date of first contact with the healthcare system was used. Where only the month was known, for instance with brain tumour diagnoses, Day 15 of the current month was selected.

### Statistical analysis

The population was analysed mainly using descriptive statistics, including percentages for categorical variables. Medians with interquartile ranges were used for continuous variables. Due to their non-Gaussian distribution, the continuous variables were compared using the nonparametric Mann–Whitney *U* test. This was applied to the null hypothesis that the distribution of complications was the same across gender, age, and diagnosis groups. A *p*-value of < 0.05 was considered significant. Analysis was performed using SPSS version 29 (IBM Corp, Armonk, NY, USA).

## RESULTS

### Population

A total of 64 patients were recruited to this study, with 42 transferred to rehabilitation directly after acute treatment in hospital and 22 admitted from home following referral from other settings (general practitioner, habilitation service, paediatric oncologist). Table III shows patient demographics.

**Table III:** Patient demographics

	N*n* (total)	TBI (%)	SCI (%)	Stroke (%)	Cerebral inf./infl. (%)	Cerebral neoplasm (%)	Anoxic brain injury (%)
*n*	64	24 (38)	8 (13)	8 (13)	10 (16)	11 (17)	3 (5)
Age at injury, mean (% of diagnosis group)							
2–6 years	12 (19)	3 (13)	2 (25)	3 (38)	0	3 (27)	1 (33)
7–10 years	10 (16)	1 (4)	2 (25)	1 (13)	2 (20)	3 (27)	1 (33)
11–14 years	19 (30)	7 (29)	2 (25)	2 (25)	4 (40)	4 (36)	0
15–17 years	23 (36)	13 (54)	2 (25)	2 (25)	4 (40)	1 (9)	1 (33)
Gender, female (%)	21 (33)	2 (8)	2 (25)	5 (63)	5 (50)	6 (55)	1 (33)
Transferred from acute care (%)	42 (66)	20 (83)	6 (75)	7 (88)	6 (60)	0	3 (100)
Admitted from home (%)	22 (34)	4 (17)	2 (25)	1 (12)	4 (40)	11 (100)	0
Length of stay, days, median (IQR)	52 (29)	55 (28)	54 (53)	54 (86)	54 (31)	44 (21)	43 (0)
Complications, yes (%)	59 (92)	21 (88)	8 (100)	8 (100)	10 (100)	10 (91)	2 (67)

TBI: traumatic brain injury, SCI: spinal cord injury, IQR: interquartile range.

All patients were discharged home, except 2 from the SCI group transferred to other hospitals, 1 for planned intervention due to complications and another for further rehabilitation.

Within the TBI group, there were 10 patients with severe injury, 6 with moderate injury, and 4 with mild injury, based on the lowest documented acute GCS score; information on the initial GCS scores for 4 patients could not be found. Patients with SCIs mainly had incomplete injuries (6/8). The 2 complete injuries were located in the thoracic segment. Of the incomplete injuries, 2 were classed as AIS C and 2 as AIS D. Two patients were unclassifiable due to young age and the consequent inability to carry out complete ASIA examinations. Most injuries were located in thoracic segments, with the exception of 2 injuries at the cervical level.

### Medical complications

Of this study population, 59 patients (92%) experienced 183 complications in total during inpatient rehabilitation, distributed across 19 complications. Within the population, 9 patients had only 1 complication, 31 had 2 or 3, 8 had 5 complications, and 5 had 6 or 7. Only 5 patients experienced no complications during their stay; of these, 3 had TBI, 1 had a brain tumour, and 1 had anoxic brain injury. These 5 patients differed from the rest in that they had a shorter LOS. Median LOS for patients who experienced complications was 55 days; this value was 35 days for those who did not experience complications, with standard deviations of 59.4 and 8.1, respectively.

There were no statistically significant differences in the number of complications between boys and girls (*p* = 0.663) or diagnostic groups (*p* = 0.768). The oldest age group (15 to 17 years) had more complications than the other age groups, with 80 complications in total; the other groups had 27 (2–6 years), 29 (7–10 years), and 48 (11–14 years) complications. There were no statistically significant differences between the age groups (*p* = 0.067).

The 3 most frequently occurring complications were sleep disturbances, pain, and constipation. All occurring complications and their distribution among diagnosis groups are outlined in Table IV. Urinary tract infections were also observed in diagnostic groups other than spinal cord injury. None of the respiratory infections were suspected to be related to aspiration.

**Table IV:** Frequency of medical complications per diagnosis group

	Total *n* (%)	TBI (*n* = 24)	SCI (*n* = 8)	Atraumatic brain injury^a^ (*n* = 32)
Sleep disturbances	34 (19)	16	3	15
Pain	28 (15)	13	4	11
Constipation	26 (14)	9	7	10
Anaemia	19 (10)	12	2	5
Infection	17 (9)	4	3	10
Spasticity	15 (8)	5	4	6
Malnutrition	14 (8)	9	1	4
Overweight	5 (3)	–	–	5
Nausea	5 (3)	–	–	5
Hormonal disorders	4 (2)	1	–	3
Pressure ulcers	3 (2)	1	2	–
Epilepsy	3 (2)	–	–	3
High blood pressure	2 (1)	1	–	1
Urinary stone formation	2 (1)	1	1	–
Hydrocephalus/CSF-related complications	2 (1)	–	–	2
Heterotopic ossification	1 (0.5)	1	–	–
Mucus formation	1 (0.5)	–	–	1
Itching^b^	1 (0.5)	–	–	1
Swollen legs^b^	1 (0.5)	–	–	1
Total number of complications (*n*)	183	73	27	83

The following complications were not reported in any patients: heart rhythm disorders, low blood pressure, blood clots, fractures, osteoporosis, scoliosis, hip dislocation, temperature control failure, cerebral haemorrhage/infarction, syringomyelia, diarrhoea, dehydration, electrolyte disturbances, urethra problems, fevers of unknown cause, and bone flap problems.

a For presentation reasons, the table consists of 3 categories, with the groups “cerebral stroke”, “cerebral infection/inflammation”, “cerebral neoplasm”, and “anoxic brain injury” merged into 1 group titled “atraumatic brain injury”.

b These were the complications registered as “other”.

TBI: traumatic brain injury, SCI: spinal cord injury.

The total median number of complications was 3; the medians per diagnosis group were 3 for TBI, 2.5 for SCI, 2.5 for cerebral stroke, 2.5 for cerebral infection/inflammation, 3 for cerebral tumour, and 4 for anoxic brain injury.

Of the 183 complications, 93 (51%) were present on admission and persisted until discharge, with 62 (34%) present on admission and resolving before discharge. A total of 30 complications occurred during stay, with half of these persisting until discharge; the other half resolved during stay.

Infection was the most frequent (*n* = 12) of these 30 complications. Other common complications were spasticity (*n* = 5), pain (*n* = 4), sleep disturbances (*n* = 2), constipation (*n* = 2), anaemia (*n* = 1), nausea (*n* = 1), urinary stone formation (*n* = 1), itching (*n* = 1), and swollen legs (*n* = 1). Of the 12 infections, 4 were urinary tract infections, 2 were respiratory tract infections, 3 were cases of oral candidiasis, 2 were wound infections related to surgery incision sites, and 1 was erythema migrans related to a tick bite. Patients with complications occurring during their stay had a marginally longer total LOS (median 56 days) than the cohort as a whole (52).

Patients admitted from home (*n* = 22) had a shorter LOS (median 44 days) and a lower number of complications (median 2.5) than those admitted directly from hospital (median LOS 62.5 days; median 3.0 complications).

### Consequences for rehabilitation

The 3 most frequently reported consequences were cancelled therapy sessions, shorter therapy sessions, and extra observational measures. Pain and infections most frequently resulted in consequences. A total of 136 (74%) complications were reported by the rehabilitation specialist (physician) as having no consequences. The 7 most frequently reported complications and their consequences are outlined in Table V.

**Table V:** Consequences of the seven most frequently occurring complications

	Total (*n* = 183)	Sleep disturbances (*n* = 34)	Pain (*n* = 28)	Constipation (*n* = 26)	Anaemia (*n* = 19)	Infection (*n* = 17)	Spasticity (*n* = 15)	Malnutrition (*n* = 14)	Other (*n* = 30)
Consequences^a^									
Cancelled therapy sessions	16	2	3	–	–	5	–	1	5
Shorter therapy sessions	16	2	6	–	–	–	–	2	6
Extra observational measures	11	–	2	–	–	5	–	1	3
Prolonged or interrupted stay	9	–	–	–	1	4	–	2	2
Delayed rehabilitation process	9	–	2	–	1	3	1	2	–
Altered process/rehabilitation goals	8	–	4	1	–	–	2	1	–
Less improvement than expected	8	–	3	–	–	–	3	1	1
Fewer planned sessions	3	–	–	–	–	1	–	1	1
None	136	31	17	25	17	8	11	9	18
Impact of consequence									
Not at all	119	25	11	24	16	10	10	6	17
Little	34	5	11	1	3	6	1	2	6
To some degree	21	3	4	1	–	2	3	2	6
Largely	9	1	2	–	–	–	1	4	1

a Possible to select more than 1.

Other complications (fewer than 6 occurrences per complication) are merged into an “other” category.

Pain, spasticity, infection, and malnutrition led to more consequences than sleep disturbances, constipation, and anaemia. Pain and infection in particular affected therapy sessions. Consequences of pain impacting rehabilitation “largely” or “to some degree” were reported in boys only (6 of 18); no consequences were reported in girls who experienced pain (10 in total). No significant differences between girls and boys in the degree of consequences were observed for the other complications. Malnutrition was the complication with the greatest impact on the rehabilitation of patients with TBI (4/6 cases impacted “largely” or “to some degree”); spasticity had consequences in the 2 oldest age groups (11–14, 15–17) only (4 of 15).

## DISCUSSION

This study evaluated the occurrence and consequences of medical complications in paediatric patients during inpatient rehabilitation following CNS injury. The majority of patients had complications on admission (*n* = 59, 92%). Half of the complications were present both on admission and on discharge. A third were present on admission only; the rest (*n* = 30) occurred during their stay. This group of 30 had a longer LOS than the cohort as a whole. The most frequently reported complications were sleep disturbances, pain, constipation, anaemia, infections, spasticity, and malnutrition. Most complications had few or no consequences for the rehabilitation process as assessed by the treating physician. However, some complications were more likely to negatively impact rehabilitation, notably malnutrition and spasticity.

Denez found that malnutrition during rehabilitation of patients with severe TBI was associated with increased LOS; higher occurrence of other complications such as pressure wounds, infections, and contractures (26); and worse functional outcomes and poorer quality of life (27). The current study found that patients with malnutrition – defined by the WHO as “deficiencies or excesses in nutrient intake, imbalance of essential nutrients or impaired nutrient utilization” (28) – had a median number of total complications of 4.2, which was greater than the overall number per patient (*n* = 3). Thus, close collaboration with nurses and nutritionists for weight and nutritional status surveillance and treatment as needed is warranted during paediatric rehabilitation. All participants identified with malnutrition in our study were already affected at the time of admission.

As spasticity from a clinical perspective can be defined as both a neurological sequela and medical complication, the distinction can be difficult to make. In the literature on complications, spasticity is sometimes noted where medication is required (10). In other papers, spasticity is not defined but listed with other effects of injury under “sequelae and complications” (5). Clinical experience has demonstrated that spasticity may improve function in some patients. In the current study, spasticity was regarded by the rehabilitation physicians as having a negative impact on patients in most cases, although they did not necessarily consider it to impact the rehabilitation process. One patient was said to have functional use of the spasticity, being able to “stand on” the spasms. For some other patients, spasticity resulted in consequences relating to altered process/rehabilitation goals and/or poorer functional levels than expected. Regular follow-ups after discharge may be beneficial for such patients, combining goal-oriented focal treatment with stretching, physiotherapy, botulinum toxin injections, and baclofen (oral or intrathecal), which is regarded as the standard treatment for patients with troublesome focal and/or regional spasticity (29).

Sleep disturbances, constipation, pain, anaemia, and infections were the most frequently occurring complications in this study. Pain and infection interfered with the therapy sessions in only a few cases. For several patients, infection resulted in a disrupted or prolonged hospital stay. Infection was also the most common complication during the stay, and its occurrence was associated with an increased LOS. According to the rehabilitation physicians, sleep disturbances, constipation, pain, and anaemia did not affect the rehabilitation process overall. However, most of these complications necessitated medications such as melatonin, laxatives, iron supplements, and paracetamol. Various studies have demonstrated that these complications cause discomfort in children with acquired CNS injury (30) and are often linked to poor quality of life outcomes (12, 31, 32). Studies on new-onset constipation in children following CNS injury are sparse, but this has been investigated in adult populations. One study on stroke patients found that new-onset constipation was associated with poorer stroke outcomes (33). Iftikhar et al. conducted a scoping review on intestinal dysfunction in TBI and found few studies on the subject (34).

In the current study, anaemia was present on admission in most cases. This complication either improved spontaneously during inpatient stay or was of low impact, and follow-up by a general practitioner was recommended after discharge.

In 8 cases, it was reported that complications led to poorer functional levels than expected. “Function” here related to everyday pursuits, characterized by the ability to participate in exercise and daily activities. Reductions in functionality were mainly related to spasticity and pain. These 8 cases comprised 4 children with TBI, 2 with SCI, and 2 with cerebral infection/inflammation.

Some patients had complications that led to prolonged or interrupted hospital stays. These were primarily complications which developed during rehabilitation. Previous studies have shown an association between the frequency of complications and increased LOS (3, 35); however, these studies do not assert causality. It remains to be determined whether complications cause longer hospital stays or longer stays cause more complications (such as healthcare-associated infections). Another possibility is that both the occurrence of medical complications and an extended length of stay reflect a generally reduced overall disability of the patient.

Although few complications impacted the rehabilitation process, those which did presented serious effects. Such complications can impact the process at both the individual and service levels. At the individual level, patients may experience worsened prognoses or need to stay longer in the hospital/be transferred to an acute care setting. These are in addition to the physical discomfort the complication may cause. At the service level, the rehabilitation team may experience disrupted sessions or increased workloads. There are also economic impacts on the institution where complications necessitate longer stays or the allocation of more resources. While this was not thematized in this study, it underscores the importance of understanding and ultimately preventing the occurrence of medical complications.

### Limitations

This was a single-centre study with relatively few participants, which reduces representativity. Only the rehabilitation physicians, not the entire MDT, evaluated the consequences of the complications. The physicians may not have had a comprehensive overview of the situation, such as the potential impact on sessions with other therapists in the MDT. Some complications may therefore have had consequences that were not recorded. There was no information on the contents of the therapy sessions and whether these were affected by the complications. Inter-rater reliability is another consideration. A total of 7 physicians evaluated the consequences of the complications; they could possibly have assessed these differently. Possible memory bias must also be considered. Patient and caregiver experiences with complications were not evaluated. Information on the effects of treatment for the medical complications was not collected in this project.

### Conclusion

Medical complications were present to a large extent during inpatient rehabilitation for the majority of patients. The physician assessments indicated that complications seldom seriously impact the rehabilitation process; however, complications such as malnutrition, infections, and spasticity appeared to impact rehabilitation to a more significant degree than others, resulting in delays in the process, increased LOS, and worsened prognoses.

More studies are required to determine the representativeness of these findings. Further research on the impact of complications on the rehabilitation process should include the entire MDT. Patient perspectives should also be considered when exploring the full scope of medical complications.

## Supplementary Material


