# Supplementary material for: MEDICAL COMPLICATIONS IN CHILDREN WITH CENTRAL NERVOUS SYSTEM INJURIES DURING INPATIENT REHABILITATION
Source: J Rehabil Med. 2026 Jun 15;58:44907. doi: 10.2340/jrm.v58.44907 (PMC13273623; doi:10.2340/jrm.v58.44907)
Supplement: Supplementary file 1 [file JRM-58-44907-s1.pdf]

MEDICAL COMPLICATIONS: ADMISSION

Date of registration: ______________________

ID no.: ____

Injury/illness: ____

____

All ICD-10 diagnoses from the acute care discharge summary:

____

____

Completed by ____

Please select below **all** complications **ongoing** during patient admission to the Sunnaas Rehabilitation Hospital. This information should be based on the emergency/acute care discharge summary and the admission interview with the patient and caregivers.

- ☐ Epilepsy
- ☐ Pain
- ☐ Infection; which ____
- ☐ Heart rhythm disorders
- ☐ High blood pressure (autonomic dysregulation)
- ☐ Low blood pressure (orthostatic hypotension)
- ☐ Blood clots (in the lungs or legs)
- ☐ Mucus formation in the lungs
- ☐ Anaemia
- ☐ Fracture
- ☐ Osteoporosis
- ☐ Scoliosis (curvature of the back)
- ☐ Heterotopic ossification (bone formation in the soft tissue close to the joints)
- ☐ Hip dislocation
- ☐ Hormonal disturbances
- ☐ Hydrocephalus
- ☐ Spasticity
- ☐ Temperature dysregulation
- ☐ Cerebral haemorrhage/infarction
- ☐ Syringomyelia/syrinx
- ☐ Constipation

- [ ] Diarrhoea
- [ ] Nausea/vomiting
- [ ] Electrolyte disturbances
- [ ] Dehydration
- [ ] Malnutrition
- [ ] Overweight
- [ ] Urinary stone formation
- [ ] Urethra problems (stricture, epididymitis)
- [ ] Bone flap problems
- [ ] Pressure ulcers
- [ ] Sleep disturbances
- [ ] Fevers of unknown origin
- [ ] Other:

For **each** complication selected, please complete the next page.

ID no.: ____

Date of registration: ____

Complication: ____

When did the complication occur? ______________________________________

Were any investigations/examinations carried out as a result of the complication? If so, which are you aware of?

Date: ________ Examination: ______________________________________

Date: ________ Examination: ______________________________________

Date: ________ Examination: ______________________________________

Date: ________ Examination: ______________________________________

What treatment(s) did the complication entail? ____________________________

Date (start/stop): ____________ Treatment: ____________________

____

Date (start/stop): ____________ Treatment: ____________________

____

MEDICAL COMPLICATIONS: DISCHARGE

Date of registration: ______________________

ID no.: ____

Injury/illness: ____

____

All ICD-10 diagnoses from the Sunnaas Rehabilitation Hospital discharge summary:

____

Completed by ____

Please select below **all** complications present during all or parts of the stay at the Sunnaas Rehabilitation Hospital.

- ☐ Epilepsy
- ☐ Pain
- ☐ Infection; which ____
- ☐ Heart rhythm disorders
- ☐ High blood pressure (autonomic dysregulation)
- ☐ Low blood pressure (orthostatic hypotension)
- ☐ Blood clots (in the lungs or legs)
- ☐ Mucus formation in the lungs
- ☐ Anaemia
- ☐ Fracture
- ☐ Osteoporosis
- ☐ Scoliosis (curvature of the back)
- ☐ Heterotopic ossification (bone formation in the soft tissue close to the joints)
- ☐ Hip dislocation
- ☐ Hormonal disturbances
- ☐ Hydrocephalus
- ☐ Spasticity
- ☐ Temperature dysregulation
- ☐ Cerebral haemorrhage/infarction
- ☐ Syringomyelia/syrinx
- ☐ Constipation
- ☐ Diarrhoea

- [ ] Nausea/vomiting
- [ ] Electrolyte disturbances
- [ ] Dehydration
- [ ] Malnutrition
- [ ] Overweight
- [ ] Urinary stone formation
- [ ] Urethra problems (stricture, epididymitis)
- [ ] Bone flap problems
- [ ] Pressure ulcers
- [ ] Sleep disturbances
- [ ] Fevers of unknown origin
- [ ] Other:

For **each** complication selected, please complete the next page.

ID no.: ____

Date of registration: ____

Complication: ____

When did the complication occur? ____

Is the complication ongoing? _________________________________

If not, when did it end? (i.e., when further examination, treatment, and monitoring were discontinued): _________________________________

Were any investigations carried out as a result of the complication? If so, what?
(Please indicate the examination location if outside Sunnaas)

Date: _________ Examination: ____

Date: _________ Examination: ____

Date: _________ Examination: ____

Date: _________ Examination: ____

What treatment(s) did the complication entail? ____________________________

Date (start/stop): ______________ Treatment: ________________________

____

Date (start/stop): ______________ Treatment: ________________________

____

COMPLICATION CONSEQUENCES

Please provide the consequences of **each** complication.

ID no.: ____

Date of registration:____

Completed by ____

This form concerns the following complication: _____________________________________

What were the consequences of this complication during stay?

- ☐ Cancelled therapy sessions
- ☐ Shorter therapy sessions
- ☐ Fewer planned sessions
- ☐ Extra observational measures. If yes, what? (e.g., continuous care, more frequent supervision, etc.) ________
- ☐ Increased physician workload
- ☐ Increased workload on other employees
- ☐ Prolonged or interrupted stay. If so, how many days prolonged/interrupted? _______
- ☐ Delayed rehabilitation process
- ☐ Altered process/rehabilitation goals
- ☐ Less improvement than expected

By your assessment, to what degree did the complication impact the overall rehabilitation process?

- ☐ Not at all
- ☐ Little
- ☐ To some degree
- ☐ Largely

Do you have any other comments regarding this complication in this patient and its impact on the rehabilitation process or outcome?
